# Prognostic Stratification of Diffuse Large B-cell Lymphoma Using Clinico-genomic Models: Validation and Improvement of the LymForest-25 Model

**DOI:** 10.1097/HS9.0000000000000706

**Published:** 2022-03-25

**Authors:** Adrián Mosquera Orgueira, Jose Ángel Díaz Arías, Miguel Cid López, Andrés Peleteiro Raíndo, Alberto López García, Rosanna Abal García, Marta Sonia González Pérez, Beatriz Antelo Rodríguez, Carlos Aliste Santos, Manuel Mateo Pérez Encinas, Máximo Francisco Fraga Rodríguez, José Luis Bello López

**Affiliations:** 1 University Hospital of Santiago de Compostela (SERGAS), Spain; 2 Health Research Institute of Santiago de Compostela, Spain; 3 University of Santiago de Compostela, Spain; 4 Fundación Jiménez Díaz, Madrid, Spain

## Abstract

Diffuse large B-cell lymphoma (DLBCL) is the most common type of non-Hodgkin lymphoma. Despite notable therapeutic advances in the last decades, 30%–40% of affected patients develop relapsed or refractory disease that frequently precludes an infamous outcome. With the advent of new therapeutic options, it becomes necessary to predict responses to the standard treatment based on rituximab, cyclophosphamide, doxorubicin, vincristine, and prednisone (R-CHOP). In a recent communication, we presented a new machine learning model (LymForest-25) that was based on 25 clinical, biochemical, and gene expression variables. LymForest-25 achieved high survival prediction accuracy in patients with DLBCL treated with upfront immunochemotherapy. In this study, we aimed to evaluate the performance of the different features that compose LymForest-25 in a new UK-based cohort, which contained 481 patients treated with upfront R-CHOP for whom clinical, biochemical and gene expression information for 17 out of 19 transcripts were available. Additionally, we explored potential improvements based on the integration of other gene expression signatures and mutational clusters. The validity of the LymForest-25 gene expression signature was confirmed, and indeed it achieved a substantially greater precision in the estimation of mortality at 6 months and 1, 2, and 5 years compared with the cell-of-origin (COO) plus molecular high-grade (MHG) classification. Indeed, this signature was predictive of survival within the MHG and all COO subgroups, with a particularly high accuracy in the “unclassified” group. Integration of this signature with the International Prognostic Index (IPI) score provided the best survival predictions. However, the increased performance of molecular models with the IPI score was almost exclusively restricted to younger patients (<70 y). Finally, we observed a tendency towards an improved performance by combining LymForest-25 with the LymphGen mutation-based classification. In summary, we have validated the predictive capacity of LymForest-25 and expanded the potential for improvement with mutation-based prognostic classifications.

## INTRODUCTION

Diffuse large B-cell lymphoma (DLBCL) is the most frequent type of aggressive B-cell lymphoma, and it exhibits a disparity of clinical outcomes. Roughly 60% of fit patients can be cured with upfront rituximab, cyclophosphamide, doxorubicin, vincristine and prednisone (R-CHOP), whereas the remaining patients develop relapsed or refractory disease, which is associated with adverse survival.^1^ Risk stratification in DLBCL has classically been based on clinical risk scores such as the International Prognostic Index (IPI), the Revised IPI, and the National Comprehensive Cancer Network IPI (NCCN-IPI).^2^ However, the discriminative power of these tools is suboptimal, as evidenced by recent studies which revealed concordance indexes (c-indexes) for overall survival in the range of 0.59–0.63. Due to these limitations, other strategies have been explored based on the identification of molecular prognostic factors. Cytogenetic data indicate that patients with *MYC* translocations and *BCL2* and/or *BCL6* translocations share a worse prognosis, and indeed these are currently classified as a different lymphoma subgroup.^3^ Studies based on gene expression data identified 3 groups of patients based on cell-of-origin (COO) status with different survival trends: germinal-center B-cell–like (GCB), activated B-cell–like (ABC), and unclassified (UNC) subgroups.^4^ In the same line, different reports indicate that those lymphomas who share a gene expression profile with double-/triple-hit or Burkitt lymphomas are also a high-risk subgroup, and these are commonly named molecular high-grade (MHG) lymphomas.^5,6^ Finally, the existence of concise prognostic subgroups based on patterns of somatic mutations has also been described, and a clear relationship between COO and mutation subgroups has also been observed.^7^ Despite such advances, there is a need to optimize and integrate these prognostic features into standardized and personalized prognostic models for real-world application.

A variety of new treatment strategies are under development for relapsed and refractory DLBCL patients. Recently, an interest to evaluate these therapies in the upfront setting of DLBCL has emerged, with a particular focus on high-risk patients. Recent data from the POLARIX trial evidenced an improvement in progression-free survival for patients with intermediate-high or high IPI (3–5) when treated with upfront polatuzumab, rituximab, cyclophosphamide, doxorubicin and prednisone versus standard R-CHOP.^8^ On the contrary, no significant differences were observed between both treatment arms for low or low-intermediate risk patients (IPI 0–2). These data indicate that substantial improvements in treatment response can be achieved with new drugs in patients who are unlikely to respond to R-CHOP. Other strategies based on anti-CD19 chimeric antigen receptor T cells or drug combinations using bispecific antibodies (eg, glofitamab) have shed promising results in the first-line setting of patients with high-risk DLBCL.^9,10^ However, there is no clinically available score capable of detecting patient subgroups who have a median 5-year overall survival <50% after R-CHOP treatment.^2^ Therefore, the growing need to identify high-risk patients at diagnosis becomes an evident unmet medical need.

In a previous effort, we developed a 54-variable model based on gene expression and a limited number of clinical variables that was capable of predicting overall survival in DLBCL patients treated with R-CHOP.^11^ More recently, we refined this prognostic model in a new cohort by incorporating data from the IPI score. This new model, named LymForest-25, contained 25 variables (19 transcripts, 5 IPI-related variables and the IPI score itself).^12^ The aim of the present study was to validate and improve LymForest-25 predictor in an external cohort of DLBCL patients. For this, we followed the following structure: (1) we evaluated if the LymForest-25 gene expression signature was prognostic and compared it with the COO and MHG classification; (2) we compared the precision of LymForest-25 gene expression signature + IPI score with that of COO and MHG classification + IPI score; and (3) we analyzed if mutation-based classifications added extra prognostic value to LymForest-25 alone. Our results indicate not only that LymForest-25 is reproducible but also that its performance is superior to the IPI score and the COO and MHG classification, particularly among younger patients.

## MATERIALS AND METHODS

### Data origin and preprocessing

Patient data was produced by the UK population-based Haematological Malignancy Research Network (https://www.hmrn.org). Pretreatment gene expression data was obtained from DLBCL biopsies pertaining to 644 patients treated with R-CHOP (Gene Expression Omnibus identification: GSE181063).^13^ Among these, 481 patients had full annotation for all the clinical and biochemical variables included in LymForest-25: age at diagnosis, baseline lactate dehydrogenase, Ann Arbor stage, number of extranodal areas affected, Eastern Cooperative Oncology Group (ECOG) score, which were used to calculate the IPI score. Gene expression data were obtained using Illumina HumanHT-12 WG-DASL V4.0 R2 gene expression bead chips. Before downstream analysis, we rank-normalized the expression estimates. Expression data for 17 of the 19 original genes included in LymForest-25 was retrieved for each patient. These genes were *PSIP1*, *ADAM12*, *SGK196*, *BCL2A1*, *LMO2*, *SULF1*, *KLHL6*, *SNHG3*, *RAB3GAP2*, *HLA-DQB2*, *CPT1A*, *SCRG1*, *ATP8A1*, *LSMEM2*/*IFRD2*, *SLC5A12*, *FNBP1* and *PDK1*. The 2 missing genes were *TRAV6* and *FAM208B*, as these were not included in the design of the gene expression chips.

### Statistical analysis

Different gene expression models were evaluated by taking either the entire expression matrix or the decomposed matrix using principal component analysis (PCA). The Cox proportional hazards model (“survival” package) was used to evaluate the performance of the different models.^14^ Time-dependent areas under the curve (AUCs), Brier scores, Akaike information criterion (AIC), and Harrel’s c-indexes were used to compare the models. For validation, bootstrapping 362+ without replacement with 500 cycles was implemented in 75% of the cohort, and the remaining 25% of samples were used for cross-validation.^15^ Bootstrapped c-indexes were computed with the “pec” package using the bootstrapping 632 method with 500 resamples.^16^ Optimism-adjusted c-indexes were calculated with the “rms” package.^17^

## RESULTS

### Cohort characteristics

Complete data for 481 DLBCL patients treated with R-CHOP was available for analysis. All cases were labeled for COO and MHG status (derived from gene expression data), and this information was codified in a single variable, that is, each case pertained to either the GCB, ABC, UNC or MHG class. A subgroup of 264 patients also had mutation-based annotation available. Four mutation-based prognostic classifications were available: (1) a 6-cluster classification developed by Lacy et al^13^ using the AIC; (2) a 7-cluster classification developed by Lacy et al,^13^ which assigns *NOTCH1* mutated lymphomas and *BCL2* and *MYC* rearranged cases to separate clusters and removes the *TET2*/*SGK1* cluster; (3) a 9-cluster prognostic classification developed by LymphGen^18^; and (4) a modified LymphGen classification that establishes a new group based on the coexistence of *MYC* rearrangement with EZB cluster membership.^18^ Baseline characteristics of the 481-patient cohort and the 264-patient subgroup were similar (Tables 1 and 2).

**Table 1:** Baseline Characteristics of DLBCL Patients Treated With R-CHOP (N = 481).

Variable	Proportion
ECOG > 2	2.50%
IPI > 2	42.41%
Raised LDH	61.54%
Ann Arbor stage > II	60.50%
Median age	65.7 y
Number of extranodal > 1	17.88%
ABC subtype	27.86%
GCB subtype	48.02%
Unclassified subtype	17.67%
MHG subtype	6.44%

ABC = activated B-cell–like; DLBCL = diffuse large B-cell lymphoma; ECOG = Eastern Cooperative Oncology Group; IPI = International Prognostic Index; LDH = lactate dehydrogenase; MHG = molecular high-grade; R-CHOP = rituximab, cyclophosphamide, doxorubicin, vincristine, and prednisone.

**Table 2:** Baseline Characteristics of the Subgroup of DLBCL Patients Treated With R-CHOP Who Had Genomic Classification Data Available for Analysis (N = 264).

Variable	Proportion
ECOG > 2	3.41%
IPI > 2	43.56%
Raised LDH	61.36%
Ann Arbor stage > II	62.50%
Median age	64.85% y
Number of extranodal > 1	17.80%
ABC subtype	28.79%
GCB subtype	46.59%
Unclassified subtype	18.56%
MHG subtype	6.06%
Lacy et al^13^ Akaike information criterion clusterization	BCL2: 21.97%
	MYD88: 14.39%
	Unclassified: 25.00%
	NOTCH2: 18.94%
	SCOS1/SGK1: 10.61%
	TET2/SGK1: 9.09%
Modified Lay et al^13^ Akaike information criterion clusterization	BCL2: 20.08%
	BCL2-MYC: 1.89%
	MYD88: 14.39%
	Unclassified: 22.73%
	NOTCH1: 3.41%
	NOTCH2: 18.18%
	SCOS1/SGK1: 10.61%
LymphGen classification	BN2: 9.85%
	BN2/N1: 0.38%
	EZB: 23.86%
	EZB/ST2: 1.14%
	MCD: 7.95%
	MCD/ST2: 0.38%
	N1: 3.03%
	Unclassified: 45.83%
	ST2: 7.58%
Modified LymphGen classification	BN2: 9.85%
	BN2/N1: 0.38%
	EZB: 22.35%
	EZB-MYC: 1.52%
	EZB/ST2: 1.14%
	MCD: 7.95%
	MCD/ST2: 0.38%
	N1: 3.03%
	Unclassified: 45.83%
	ST2: 7.58%

ABC = activated B-cell–like; DLBCL = diffuse large B-cell lymphoma; ECOG = Eastern Cooperative Oncology Group; IPI = International Prognostic Index; LDH = lactate dehydrogenase; MHG = molecular high-grade; R-CHOP = rituximab, cyclophosphamide, doxorubicin, vincristine, and prednisone.

### Evaluation of the LymForest-25 gene expression signature

**Figure 1.**
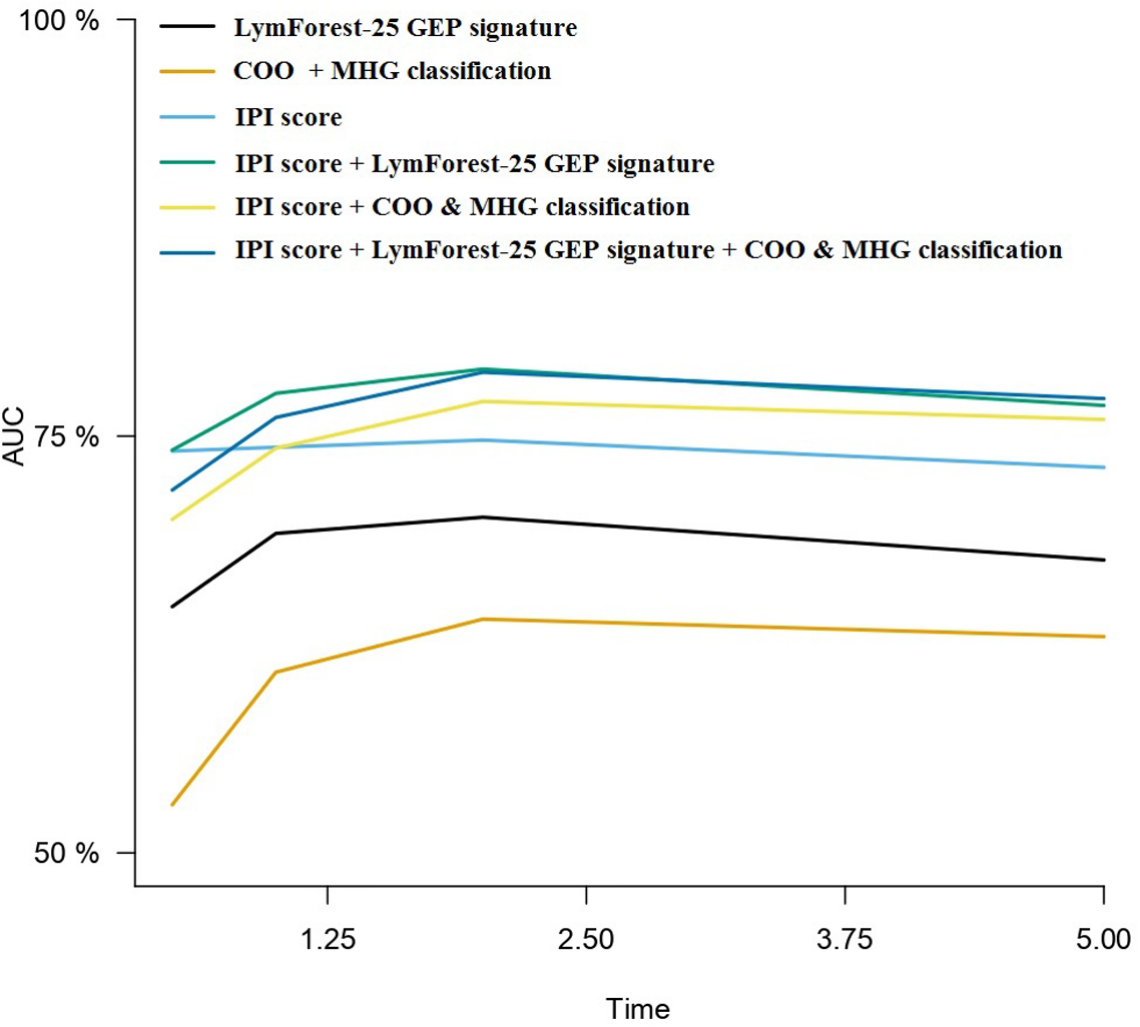
**Representation of time** **-dependent AUCs of the different models evaluated in the whole DLBCL cohort (N = 481).** AUC = area under the curve; COO = cell-of-origin; DLBCL = diffuse large B-cell lymphoma; GEP = gene expression profiling; IPI = International Prognostic Index; MHG = molecular high-grade.

Initially, we evaluated the performance of survival models taking either the whole LymForest-25 gene expression matrix (17 genes) or a decomposed matrix obtained using PCA. According to AIC and bootstrapped c-indexes, the model using the first 4 principal components (PC4) was superior to the remaining models (Figure 1; Table 3). Therefore, we evaluated this model in comparison with the COO + MHG classification. As indicated in Table 3, time-dependent AUCs and Brier scores revealed that the LymForest-25 PC4 model was substantially superior to the COO + MHG at all time points evaluated, and the model was also superior in terms of Brier score, AIC, and bootstrapped c-index (Suppl. Table S1).

**Table 3:** Evaluation of the 17-gene Expression Signature Using Whole Gene Expression Data and Principal Components.

Model	AIC	C-index
17 genes	2290	60.0
PC1	2307	54.2
PC1 + PC2	2284	60.9
PC1 + PC2 + PC3	2279	60.7
PC1 + PC2 + PC3 + PC4	2275	61.7
PC1 + PC2 + PC3 + PC4 + PC5	2277	61.5

Bootstrapped c-indexes and Akaike information criteria are provided. Results were obtained from the whole cohort of DLBCL patients treated with R-CHOP (N = 481).

AIC = Akaike information criterion; c-index = concordance index; DLBCL = diffuse large B-cell lymphoma; PC = principal component; R-CHOP = rituximab, cyclophosphamide, doxorubicin, vincristine, and prednisone.

### Optimization of LymForest-25 using the IPI score

**Figure 2.**
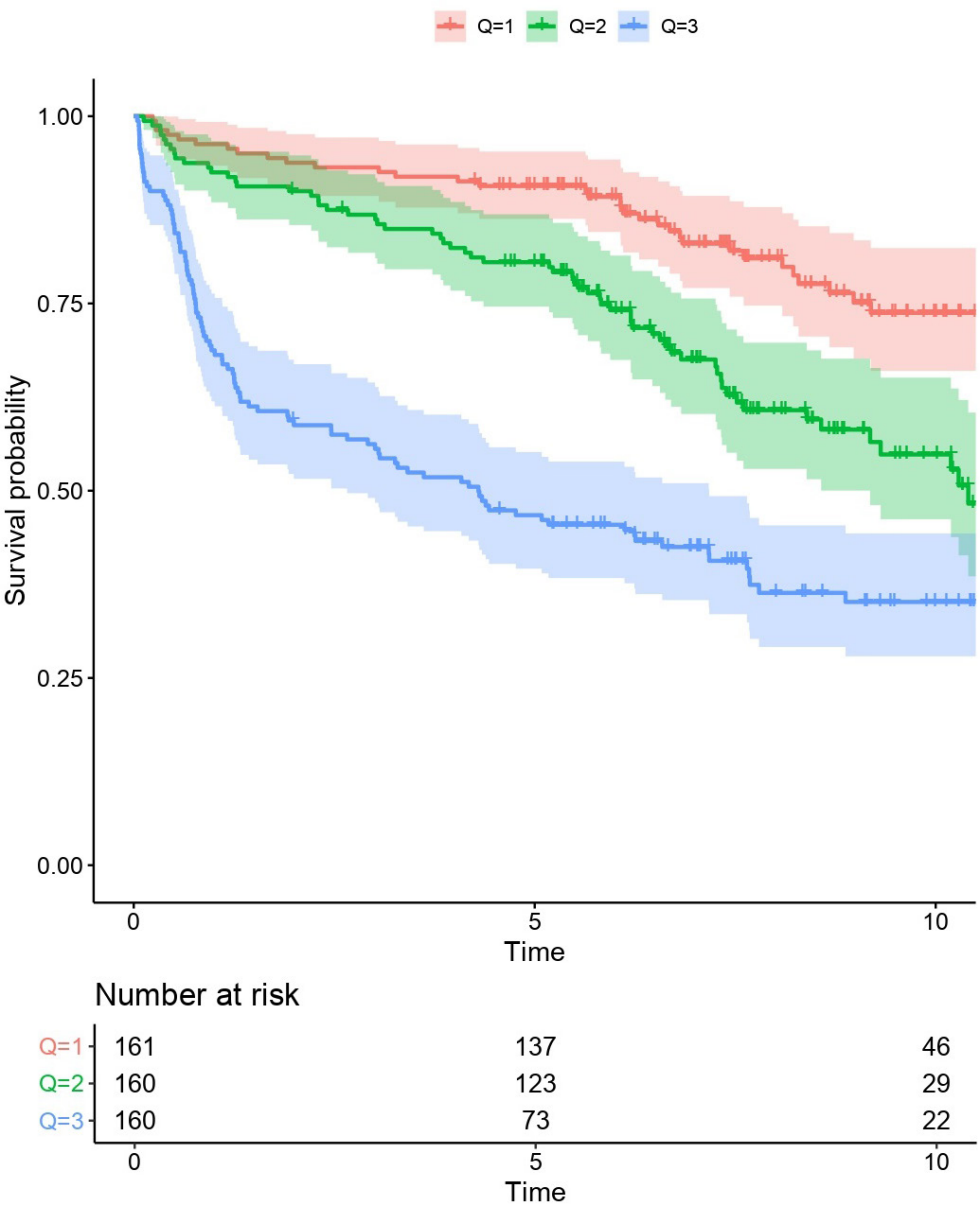
**Outcome of patients stratified by to tertiles of expected survival according to LymForest-25 gene expression signature and the IPI score (cox regression).** IPI = International Prognostic Index.

We evaluated the performance of the LymForest-25 gene expression signature (PC4) combined with the IPI score (Figure 2). The results indicate that the new signature was substantially superior to the IPI score combined with COO + MHG classification at all time points evaluated during the first 5 years after diagnosis, both in terms of Brier scores and time-dependent AUCs (Figure 1; Table 4). Importantly, the model achieved an AUC of 78.73% for predicting 2-year survival. Considering these metrics, no substantial benefit was obtained by integrating the PC4 signature with the COO + MHG classification into the same model.

**Table 4:** Time-dependent AUCs and Brier Scores of the Different Survival Models.

Model	Time (y)	AUC	Brier Score
17 genes (PC1 + PC2 + PC3 + PC4)	0.5	65.11	0, 063
17 genes (PC1 + PC2 + PC3 + PC4)	1	69.12	0, 113
17 genes (PC1 + PC2 + PC3 + PC4)	2	69.79	0, 142
17 genes (PC1 + PC2 + PC3 + PC4)	5	67.21	0, 183
COO + MHG	0.5	52.78	0, 066
COO + MHG	1	60.69	0, 119
COO + MHG	2	64.14	0, 150
COO + MHG	5	63.27	0, 190
IPI score	0.5	74.70	0, 061
IPI score	1	74.27	0, 109
IPI score	2	74.57	0, 136
IPI score	5	72.83	0, 173
IPI score + 17 genes (PC1 + PC2 + PC3 + PC4)	0.5	74.56	0, 058
IPI score + 17 genes (PC1 + PC2 + PC3 + PC4)	1	77.48	0, 101
IPI score + 17 genes (PC1 + PC2 + PC3 + PC4)	2	78.73	0, 124
IPI score + 17 genes (PC1 + PC2 + PC3 + PC4)	5	76.45	0, 160
IPI score + COO + MHG	0.5	70.38	0, 062
IPI score + COO + MHG	1	74.11	0, 107
IPI score + COO + MHG	2	76.98	0, 130
IPI score + COO + MHG	5	75.95	0, 162
IPI score + COO + MHG + 17 genes (PC1 + PC2 + PC3 + PC4)	0.5	72.05	0, 059
IPI score + COO + MHG + 17 genes (PC1 + PC2 + PC3 + PC4)	1	75.91	0, 102
IPI score + COO + MHG + 17 genes (PC1 + PC2 + PC3 + PC4)	2	78.57	0, 124
IPI score + COO + MHG + 17 genes (PC1 + PC2 + PC3 + PC4)	5	76.96	0, 160

Evaluated time points were 6 mo and 1, 2, and 5 y after diagnosis. Results were obtained from the whole cohort of DLBCL patients treated with R-CHOP (N = 481).

AUC = area under the curve; COO = cell-of-origin; DLBCL = diffuse large B-cell lymphoma; IPI = International Prognostic Index; MHG = molecular high-grade; PC = principal component; R-CHOP = rituximab, cyclophosphamide, doxorubicin, vincristine, and prednisone.

### Subanalysis within cell-of-origin and molecular high-grade subgroups

Our previous work with LymForest-25 revealed that COO classification did not provide any additional prognostic information when integrated with the new classifier, and so this variable was not included in the final model.^12^ In order to inspect this effect here, we evaluated the performance of the LymForest-25 gene expression signature (PC4) within each COO group and in patients with lymphomas classified as MHG. Time-dependent AUC confirmed that the novel gene expression signature was prognostic in all cases but with remarkable differences between subgroups (Table 5). For example, a substantial improvement in risk prediction was observed in unclassified COO DLBCL, with AUCs >70% at all time points evaluated. Predictions within the ABC and GCB subgroups were less accurate but nonetheless substantially superior to 50%. Interestingly, in these groups, the signature was more precise in the identification of early events (6 mo and 1 y) than delayed ones (2 and 5 y). On the contrary, the signature was less accurate to predict early events than late ones in the MHG subgroup, although the interpretation of this finding should be taken cautiously due to the small sample size of this class (N = 31).

**Table 5:** Time-dependent AUCs and Brier Scores for the Different Survival Models Within the ABC, GCB, Unclassified and MHG Groups.

Model	Time (y)	ABC		GCB		Unclassified		MGH	
		AUC	Brier Score	AUC	Brier Score	AUC	Brier Score	AUC	Brier Score
17 genes (PC1 + PC2 + PC3 + PC4)	0.5	64.40	0, 069	61.42	0, 058	74.41	0, 072	53.19	0, 106
17 genes (PC1 + PC2 + PC3 + PC4)	1	58.06	0, 162	61.37	0, 085	78.47	0, 108	49.48	0, 245
17 genes (PC1 + PC2 + PC3 + PC4)	2	56.62	0, 216	59.18	0, 103	78.81	0, 114	58.22	0, 264
17 genes (PC1 + PC2 + PC3 + PC4)	5	53.21	0, 252	55.81	0, 151	73.69	0, 163	56.04	0, 287

Evaluated time points were 6 mo and 1, 2, and 5 y after diagnosis.

ABC = activated B-cell–like; AUC = area under the curve; GCB = germinal-center B-cell–like; MHG = molecular high-grade; PC = principal component.

### Model performance in younger and older patients

We evaluated the performance of the best prognostic models separately for younger (≤70 y old, N = 302) and older (>70 y old, N = 179) patients. We confirmed the superiority of the LymForest-25 gene expression signature compared to the COO + MHG classification in both subgroups (Table 6; Suppl. Table S2). However, notable differences emerged. Firstly, we observed that mortality prediction accuracy was inferior in older patients for all models, including the IPI score. Secondly, although both gene expression-based signatures retained individual prognostic accuracy among older patients, time-dependent AUCs revealed that their integration with the IPI score did not provide any improvement to the IPI score alone. Therefore, these results suggest that the power of molecular predictors of survival appears to be partially limited to younger patients.

**Table 6:** Time-dependent AUCs and Brier Scores for the Different Survival Models in Younger (≤70 Years) and Older (>70 Years) Patients.

Model	Time (y)	≥70 y (N = 179)		<70 y (N = 302)	
		AUC	Brier Score	AUC	Brier Score
17 genes (PC1 + PC2 + PC3 + PC4)	0.5	54.50	0, 099	75.42	0, 044
17 genes (PC1 + PC2 + PC3 + PC4)	1	64.85	0, 145	71.92	0, 095
17 genes (PC1 + PC2 + PC3 + PC4)	2	63.87	0, 181	74.01	0, 117
17 genes (PC1 + PC2 + PC3 + PC4)	5	65.13	0, 221	68.08	0, 154
COO + MHG	0.5	45.11	0, 103	58.17	0, 045
COO + MHG	1	54.23	0, 158	63.89	0, 098
COO + MHG	2	56.81	0, 196	67.01	0, 121
COO + MHG	5	54.76	0, 243	66.71	0, 155
IPI score	0.5	68.75	0, 098	78.00	0, 042
IPI score	1	73.43	0, 147	73.93	0, 091
IPI score	2	70.93	0, 182	76.11	0, 110
IPI score	5	68.50	0, 223	74.06	0, 142
IPI score + 17 genes (PC1 + PC2 + PC3 + PC4)	0.5	60.30	0, 096	81.93	0, 039
IPI score + 17 genes (PC1 + PC2 + PC3 + PC4)	1	70.01	0, 140	78.06	0, 084
IPI score + 17 genes (PC1 + PC2 + PC3 + PC4)	2	69.56	0, 173	81.30	0, 100
IPI score + 17 genes (PC1 + PC2 + PC3 + PC4)	5	68.96	0, 213	77.80	0, 133
IPI score + COO + MHG	0.5	59.42	0, 100	74.88	0, 041
IPI score + COO + MHG	1	68.64	0, 147	75.01	0, 088
IPI score + COO + MHG	2	68.79	0, 180	79.48	0, 105
IPI score + COO + MHG	5	65.98	0, 225	78.40	0, 133
IPI score + COO + MHG + 17 genes (PC1 + PC2 + PC3 + PC4)	0.5	57.31	0, 099	78.36	0, 039
IPI score + COO + MHG + 17 genes (PC1 + PC2 + PC3 + PC4)	1	68.11	0, 144	76.40	0, 085
IPI score + COO + MHG + 17 genes (PC1 + PC2 + PC3 + PC4)	2	67.60	0, 177	80.89	0, 100
IPI score + COO + MHG + 17 genes (PC1 + PC2 + PC3 + PC4)	5	66.48	0, 221	78.82	0, 131

Evaluated time points were 6 mo and 1, 2, and 5 y after diagnosis.

AUC = area under the curve; COO = cell-of-origin; IPI = International Prognostic Index; MHG = molecular high-grade; PC = principal component.

### Integration with prognostic features based on mutation clusters

Prognostic mutation-based classifications were originally evaluated in a subgroup of 264 patients. Therefore, we set out to understand how LymForest-25 performance could be improved with such prognostic features. Due to the existence of very small clusters in these mutation-based classifications, we were unable to perform bootstrapping to compare time-dependent AUCs and Brier scores. However, we could evaluate AICs and optimism-adjusted c-indexes (Suppl. Table S3). Considering c-indexes, no relevant improvement was observed with any of the mutation-based classifications. AICs indicated that LymForest-25 was superior to its integration with the models developed by Lacy et al,^13^ the modified model by Lacy et al^13^ and the LymphGen model. On the contrary, a difference in more than 2 AIC units was observed after the introduction of the modified LymphGen model, which points towards a modest but significant improvement.

## DISCUSSION

A growing need exists to develop precise prognostic models in DLBCL patients treated with R-CHOP. Such models must incorporate traditional prognostic variables (ie, IPI score) with the different layers of molecular complexity of DLBCL that have been discovered in the last years. Such an approach should enable a more robust identification of high-risk patients from the moment of diagnosis. Currently, clinical and molecular risk scores rely on patient subgrouping, which create suboptimal predictions for individual cases. For example, according to a recent report, the high-intermediate and high-risk subgroups of the best clinical risk score (NCCN-IPI) have 5-year overall survival rates of 62.7% and 49.0%, respectively.^2^ These findings imply that a major part of such “high risk” patients will be still alive at this time point. In a similar line, 8.2% of the high-risk *MYD88*^mut^ DLBCL patients have a GCB COO status, which is a maker associated with favorable outcomes. On the contrary, 10.0%, 6.2%, and 2.7% of patients assigned to the low-risk *TET2*/*SGK1*, *SCOS1*/*SGK* and *BCL2* clusters, respectively, have an ABC COO status, which is a predictor of short survival. Remarkably, up to 27% and 21.6% of patients in the same study were assigned to an unclassified subgroup with intermediate prognosis according to the mutational and COO classifications, respectively.^13^ Altogether, these findings highlight the need for an integrated model capable of accommodating the prognostic complexity of DLBCL and making optimal survival predictions for each individual case.

In this study, we have evaluated a prognostic model based on clinical and genomic data that we presented in recent publications.^11,12^ This predictor outperforms currently established molecular subtypes of DLBCL, such as the COO and MHG status, and improves substantially the prognostic capacity of the IPI score. The model retained important prognostic information within each COO subgroup and in patients with high-risk lymphomas defined by a gene expression signature that resembles Burkitt lymphoma (MHG group). Remarkably, the best prediction accuracy was observed in patients whose COO status was unclassified. Furthermore, we have observed that a promising strategy to optimize these predictions relies on the integration of LymForest-25 with the modified LymphGen mutation-based classification.

Another important conclusion of our modeling strategy points towards a limitation of molecular data in the survival prediction of older patients (>70 y). Treatment dose intensity can be largely conditioned by comorbidities and patient’s frailty that are not adequately reflected by age and the ECOG status. In this case, improvements might be achieved by incorporating into the model the results of scores based on integrated geriatric assessment. Indeed, the benefits of this strategy in DLBCL have been extensively studied by different groups.^19,20^

The present work is in line with other efforts that highlight the importance of advanced data analysis in lymphoma prognostication. Recently, Zaccaria et al^21^ used machine learning techniques to evaluate data from a mantle cell lymphoma phase III trial developed by the Fondazione Italiana Linfomi. Briefly, they applied unsupervised clustering techniques to baseline clinical data, identifying 3 groups of patients with different survival. Importantly, they validated these findings in 2 independent cohorts. The new prognostic score retained similar prognostic information as other prognostic scores in the field but produced more balanced clusters. Notably, this score also revealed a reduced performance among old patients. On the contrary, our strategy aims for a more complex evaluation of lymphoma by including clinical scores and molecular profiles to directly model survival. We observed that this strategy is superior to “standard” classifications within the first 5 years after diagnosis, which is the temporal window where lymphoma-related mortality is concentrated.^22^ As a drawback, our method requires the application of relatively complex molecular techniques that are not readily available to most centers.

Our study protocol had some limitations. Firstly, not all the 19 transcripts of the LymForest-25 classifier were measured in the present cohort, and our evaluation had to rely on 17 transcripts. Even so, the prognostic accuracy of this transcriptomic signature added substantial improvements to the IPI score, and it performed substantially better than those models based on the COO + MHG status. Secondly, although we used bootstrapping to validate our results, these findings should be verified in additional cohorts.

In conclusion, we have confirmed the validity of a new risk stratification model in DLBCL. This tool, named LymForest-25, exhibited an superior performance compared with the MHG and COO classifications, and it was particularly useful to risk-stratify younger patients. Additionally, we provide preliminary evidence that the integration with the LymphGen mutation classification can enhance the performance of the model. Therefore, the precise integration of these approaches emerges as a promising strategy in order to achieve highly accurate survival predictions in the field of DLBCL.

## AUTHOR CONTRIBUTIONS

AMO had the idea, designed the article, analyzed the data, and wrote the article. JADA, MCL, APR, ALG, RAG, MSGP, BAR, CAS, MMPE, MFFR, and JLBL reviewed the results, suggested modifications and approved the final publication.

## DISCLOSURES

AMO reports honoraria for lectures and participation in advisory boards from Janssen, Takeda, Abbey, Amgen, Novartis, Gilead and AstraZeneca; research grants from Roche, Pfizer, and Celgene-BMS and funds for conference organization from Jassen, Takeda, Abbey, Amgen, Novartis, Gilead, Roche, Bristol-Myers-Squibb, GlaxoSmithKline, Incyte and Pfizer. All the other authors have no conflicts of interest to disclose.

## Supplementary Material


