# Supplementary material for: Prognostic Stratification of Diffuse Large B-cell Lymphoma Using Clinico-genomic Models: Validation and Improvement of the LymForest-25 Model
Source: Hemasphere. 2022 Mar 25;6(4):e706. doi: 10.1097/HS9.0000000000000706 (PMC8984321; doi:10.1097/HS9.0000000000000706)
Supplement: Supplementary file 1 [file hs9-6-e706-s001.pdf]

Model	C-index	AIC
**17 genes (PC1+PC2+PC3+PC4)**	61.6	2.275
**COO + MHG**	59.8	2.280
**IPI score**	66.3	2.239
**IPI score + 17 genes (PC1+PC2+PC3+PC4)**	68.4	2.216
**IPI score + COO + MHG**	69.0	2.217
**IPI score + COO + MHG + 17 genes (PC1+PC2+PC3+PC4)**	69.1	2.211
