# Supplementary material for: Prognostic Stratification of Diffuse Large B-cell Lymphoma Using Clinico-genomic Models: Validation and Improvement of the LymForest-25 Model
Source: Hemasphere. 2022 Mar 25;6(4):e706. doi: 10.1097/HS9.0000000000000706 (PMC8984321; doi:10.1097/HS9.0000000000000706)
Supplement: Supplementary file 2 [file hs9-6-e706-s002.pdf]

	>=70 years		<70 years	
**Model**	**AIC**	**C-index**	**AIC**	**C-index**
**17 genes (PC1+PC2+PC3+PC4)**	964	60.1	1.011	60.9
**COO + MHG**	975	55.2	1.008	60.3
**IPI score**	970	60.1	972	69.6
**IPI score + 17 genes (PC1+PC2+PC3+PC4)**	961	61.9	966	70.4
**IPI score + COO + MHG**	968	60.9	963	72.1
**IPI score + COO + MHG + 17 genes (PC1+PC2+PC3+PC4)**	966	61.2	963	71.4
