# Supplementary material for: Prognostic Stratification of Diffuse Large B-cell Lymphoma Using Clinico-genomic Models: Validation and Improvement of the LymForest-25 Model
Source: Hemasphere. 2022 Mar 25;6(4):e706. doi: 10.1097/HS9.0000000000000706 (PMC8984321; doi:10.1097/HS9.0000000000000706)
Supplement: Supplementary file 3 [file hs9-6-e706-s003.pdf]

Model	C-index	AIC
**LymForest (IPI score + 4 PC)**	70.37	1.161
**LymForest (IPI score + 4 PC)<br>+<br>Lacy et al. (2020)**	70.49	1.163
**LymForest (IPI score + 4 PC)<br>+<br>Modified Lacy et al. (2020)**	69.86	1.163
**LymForest (IPI score + 4 PC)<br>+<br>LymphGen**	69.50	1.165
**LymForest (IPI score + 4 PC)<br>+<br>Modified LymphGen**	70.63	1.158
